# A Systematic Review of WTA-WTP Disparity for Dental Interventions and Implications for Cost-Effectiveness Analysis

**DOI:** 10.3390/healthcare8030301

**Published:** 2020-08-26

**Authors:** Pedram Sendi, Arta Ramadani, Nicola U. Zitzmann, Michael M. Bornstein

**Affiliations:** 1 Department of Oral Health and Medicine, University Center for Dental Medicine Basel, University of Basel, 4058 Basel, Switzerland; arta.ramadani91@gmail.com (A.R.); michael.bornstein@unibas.ch (M.M.B.); 2 Institute for Clinical Epidemiology, Basel University Hospital, 4031 Basel, Switzerland; 3 Department of Reconstructive Dentistry, University Center for Dental Medicine Basel, University of Basel, 4058 Basel, Switzerland; n.zitzmann@unibas.ch

**Keywords:** health services research, economic evaluation, public health, dental implants, water fluoridation, dentistry, contingent valuation, willingness to pay

## Abstract

Cost-effectiveness analysis is widely adopted as an analytical framework to evaluate whether health care interventions represent value for money, and its use in dentistry is increasing. Traditionally, in cost-effectiveness analysis, one assumes that the decision maker’s maximum willingness to pay (WTP) for health gain is equivalent to his minimum willingness to accept (WTA) monetary compensation for health loss. It has been documented in the literature that losses are weighted higher than equivalent gains, i.e., that WTA exceeds WTP for the same health condition, resulting in a WTA/WTP ratio greater than 1. There is a knowledge gap of published WTA/WTP ratios for dental interventions in the literature. We therefore conducted a (i) systematic review of published WTA-WTP estimates in dentistry (MEDLINE, Web of Science, Cochrane Library, London, UK) and (ii) a patient-level analysis of WTA/WTP ratios of included studies, and (iii) we demonstrate the impact of a WTA-WTP disparity on cost-effectiveness analysis. Out of 55 eligible studies, two studies were included in our review. The WTA/WTP ratio ranged from 2.58 for discontinuing water fluoridation to 5.12 for mandibular implant overdentures, indicating a higher disparity for implant rehabilitations than for dental public health interventions. A WTA-WTP disparity inflates the cost-effectiveness of dental interventions when there is a substantial risk of both lower costs and health outcomes. We therefore recommend that in these cases the results of cost-effectiveness analyses are reported using different WTA/WTP ratios in a sensitivity analysis.

## 1. Introduction

Cost-effectiveness analysis is widely adopted as an analytical framework to evaluate whether health care interventions represent value for money [1]. The rationale behind cost-effectiveness analysis is to increase the efficiency of the allocation of scarce health care resources [1]. The number of published cost-effectiveness analyses in dentistry has substantially increased over the last 40 years [2,3]. The analytical tool of cost-effectiveness analysis is the incremental cost-effectiveness ratio (ICER), which represents the ratio of the difference in costs between two interventions (incremental costs) by the difference in effects (incremental effects) [4]. If the ICER is below a threshold value (i.e., the ceiling ratio), the intervention is considered as cost-effective and is adopted, while an ICER above the threshold value is considered as cost-ineffective, and such a health care intervention is therefore rejected. The ceiling ratio is often interpreted as the decision maker’s maximum willingness-to-pay (WTP) per unit of health outcome [5,6]. While this interpretation holds true for interventions that increase both health outcomes and costs, there may be situations where an intervention reduces both health outcomes and costs. In these cases, the ceiling ratio has a different interpretation and represents the decision maker’s minimum willingness-to-accept (WTA) compensation in monetary terms for relinquishing health outcomes [7].

Since cost and effects of health interventions are stochastic, i.e., subject to uncertainty, a number of methods have been developed to describe this uncertainty surrounding the joint distribution of incremental costs and effects [8,9,10,11,12,13,14]. The most widely used framework for handling uncertainty in cost-effectiveness analysis is the cost-effectiveness acceptability curve [8,15]. The cost-effectiveness acceptability curve summarizes this uncertainty by estimating the probability that an intervention is cost-effective for a wide range of ceiling ratios. However, current methods for handling uncertainty in cost-effectiveness analysis assume that the maximum WTP for an intervention is equal to the minimum WTA to forgo health benefits [16]. It has been documented in the broader economic and health economic literature, that losses are valued higher than equivalent gains, i.e., that WTA exceeds WTP for the same health condition and, therefore, results in a WTA/WTP ratio greater than 1 [7,17,18,19]. Since a disparity in WTA and WTP may substantially impact how the results of a cost-effectiveness analysis ought to be interpreted, this is of importance for the dental community evaluating the cost-effectiveness of dental interventions. Handling uncertainty in cost-effectiveness analysis without considering a WTA/WTP ratio greater than 1 may lead to biased estimates of the economic value of a dental health intervention. To the best of our knowledge, there is an absence of published WTA/WTP ratios for dental interventions in the literature.

The objective of the present study is therefore (i) to systematically review studies of dental interventions that report both WTP and WTA values, (ii) to estimate WTA/WTP ratios for dental interventions using patient level data, and (iii) to demonstrate the impact of a WTP-WTA disparity on the cost-effectiveness of dental health care.

## 2. Methods

### 2.1. Systematic Review

**Figure 1:**
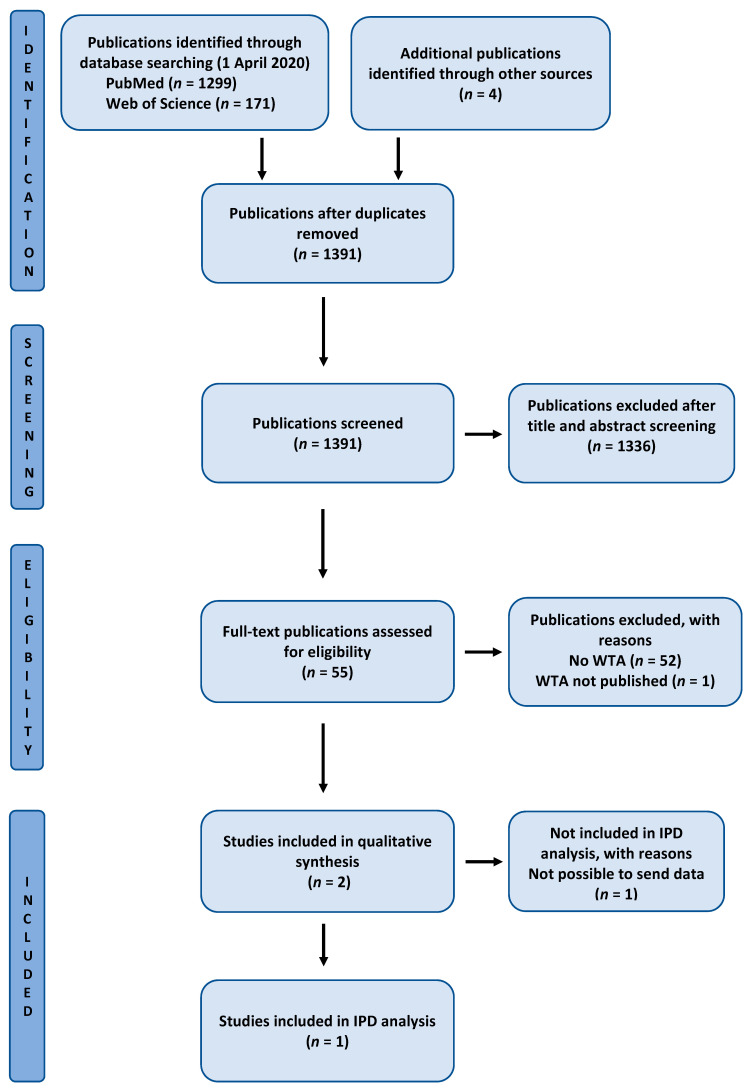
PRISMA (Preferred Reporting Items for Systematic Reviews and Meta-Analyses) flow diagram. IPD, individual participant data; WTA, willingness to accept.

We searched the databases MEDLINE via PubMed and Web of Science on 1 April 2020, using a combination of Medical Subject Headings (MeSH in PubMed) and general search terms (PubMed and Web of Science). The search strategy for Web of Science included the query: (“Willingness to pay” OR WTP OR “Willingness to accept” OR WTA OR “Contingent valuation” OR “Conjoint analysis” OR “Cost benefit analysis” OR “Discrete choice experiment” OR “Monetary value”) AND Topic: “dent*”. The search strategy for PubMed included the query: (“Willingness to pay” OR WTP OR “Willingness to accept” OR WTA OR “Contingent valuation” OR “Conjoint analysis” OR “Cost benefit analysis” OR “Discrete choice experiment” OR “Monetary value”) AND (Dentistry [MeSH] or “dent *”). In addition, we hand-searched the references of a published systematic review on WTP estimates for dental interventions for eventual reporting of WTA estimates (Figure 1, “other sources”) [20]. We also searched the Cochrane Library (www.cochranelibrary.com) and the PROSPERO database (www.crd.york.ac.uk/prospero) for published or eventual ongoing studies that assessed both WTP or WTA of dental interventions on 11 May 2020. 

After exclusion of duplicates, titles and abstracts were screened by two reviewers (Arta Ramadani) and (Pedram Sendi) and further considered using the following eligibility criteria: publications in English or German, empirical studies (as opposed to conceptual/theoretical papers), reporting WTP or WTA estimates for a dental service or good, elicited in the same or comparable group, full-text available. If from the abstract and titles eligibility was not clear, the full-text article was screened to assess eligibility and resolved by discussion between reviewers. Eligible studies were further considered in a full-text analysis and included if both WTP and WTA estimates were reported for the same dental service or good. For each included article, we extracted the respective WTA/WTP ratio by dividing mean WTA by mean WTP (ratio of means) for the same intervention (Table 1). In addition, the following study characteristics were collected: dental intervention, sample size, within- vs. between-subject design, study design, elicitation method, 1st author, year of publication, country.

### 2.2. Analysis of Patient-Level Data

In addition to the extracted WTA/WTP ratio (ratio of means) from the data provided in the included publications, patient level data to estimate a confidence interval for the WTA/WTP ratio in each study were used by estimating the WTA/WTP ratio for each patient (mean WTA/WTP ratio, see Table 1). We did not combine the results of the different studies as the WTA/WTP ratio may strongly depend on the dental service or good provided, and we therefore sought to provide separate estimates of the WTA/WTP ratio for each dental health intervention. We first conducted a complete case analysis for patients providing both WTA and WTP values and estimated a 95% confidence interval for the mean WTA/WTP ratio with non-parametric bootstrapping using 10,000 samples. Since patients may eventually provide an infinite large number for WTA values or may not be willing to engage in the WTA/WTP elicitation procedure, these patients were considered as “protesters” and the respective values as missing in the dataset [21]. We therefore further analyzed the data by replacing missing values using multiple imputation by chained equations, i.e., fully conditional specification using auxiliary variables to improve the estimation of the WTA/WTP ratio [22]. We generated for each included study 1000 imputed datasets and estimated a pooled mean WTA/WTP ratio and the respective 95% confidence interval using Rubin’s rule [23]. The statistical analyses were performed using R and RStudio (Version 1.2.1335, RStudio, Inc., Boston, MA, USA).

## 3. Results

Databases were searched on 1 April 2020 (MEDLINE and Web of Science), and 11 May 2020 (Cochrane Library and PROSPERO database). A total of 1474 records were identified, and after removal of duplicates, 1391 remained for title and abstract screening. We included 55 articles in full-text screening, 2 remained for qualitative and quantitative analysis, after the exclusion of 53 papers with reasons (for exclusion details see Figure 1). The eligible studies that were excluded are listed in the Supplementary Materials with reasons for exclusion. Patient-level data for further analysis were only available for one study. Figure 1 displays the PRISMA (Preferred Reporting Items for Systematic Reviews and Meta-Analyses) flow diagram. 

Table 1 displays the descriptive characteristics and the extracted WTA/WTP ratios of the studies included in the systematic review [24,25]. The study by Dixon and Shackley [25] was conducted in the UK and assessed both WTP and WTA values in a group of individuals who were against water fluoridation. They were asked how much they would be willing to pay to remove fluoride from water (WTP format) and how much they would require in compensation to accept water fluoridation (WTA format). Individual participant data were not available; however, the WTA/WTP ratio (ratio of means) extracted from the publication for discontinuing water fluoridation was 2.58 (Table 1). Sendi et al. [24] assessed the WTP for two interforaminal implants to retain a mandibular complete denture in patients who all underwent implant surgery previously. The same patients were also asked how much they would require in monetary compensation (WTA) to accept to be in the same oral health state than before implant surgery; the WTA/WTP ratio was 5.12 (95% CI: 2.61–7.62) (Table 1).

## 4. Implications for Cost-Effectiveness Analysis

The incremental cost-effectiveness ratio (ICER) of two treatment strategies is estimated by dividing the incremental costs (ΔC) by the incremental effects (ΔE)

(1) $$\mathrm{ICER}\;=\;\frac{\Delta C}{\Delta E}$$

**Figure 2:**
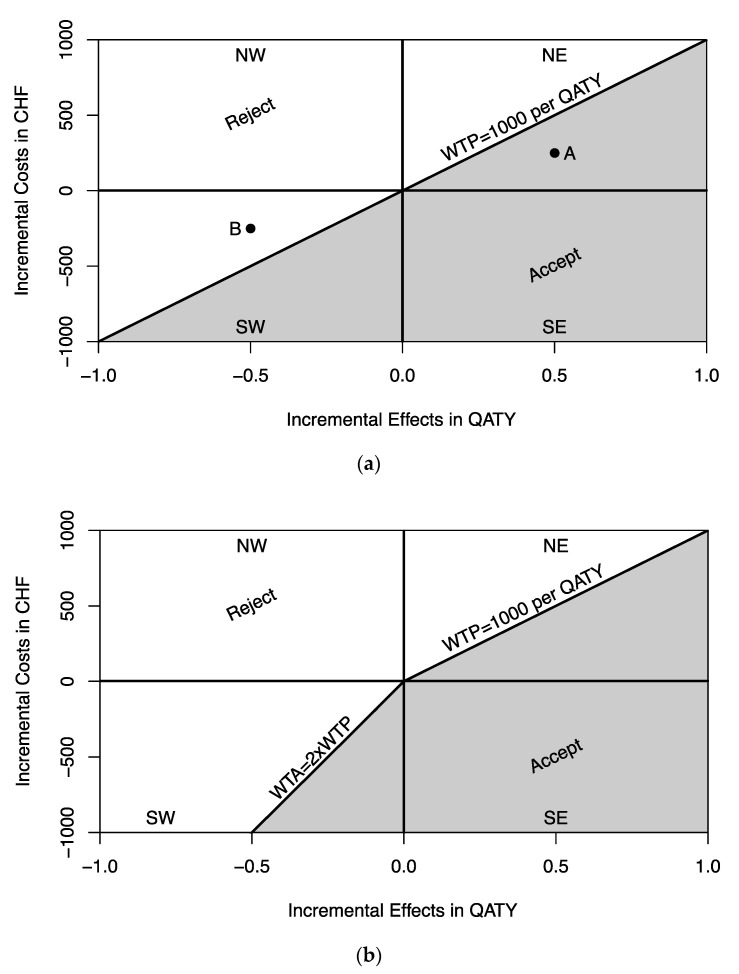
(**a**) Cost-effectiveness plane (WTP = WTA). (**b**) Cost-effectiveness plane (WTP = 2 × WTA). WTA, willingness to accept; WTP, willingness to pay; ceiling ratio WTP = WTA = 1000 $/QATY in (**a**); NW, North-West; SW, South-West; NE, North-East; SE, South-East. In (**a**) Programs A and B have the same ICER of 500 $/QATY; grey shaded area indicates programs with an acceptable ICER in (**a**,**b**).

The results of a cost-effectiveness analysis can be graphically presented on the cost-effectiveness plane (CEP) as shown in Figure 2a [26]. The CEP entails four quadrants (Figure 2a). The North-East quadrant (NE) describes outcomes where ΔC and ΔE are positive (i.e., higher costs and health effects). In the NE quadrant, one typically estimates the ICER. The South-East (SE) quadrant describes situations where ΔC is negative and ΔE positive (i.e., cost savings and health gains). Outcomes in the SE quadrant are always accepted (i.e., dominance), and the ICER is therefore not estimated. The South-West (SW) quadrant describes situations where both ΔC and ΔE are negative (i.e., cost savings and health loss). In the SW quadrant, one also estimates the ICER. In the North-West (NW) quadrant, ΔC is positive and ΔE negative (i.e., higher costs and health loss). Outcomes in the NW quadrant are always rejected (i.e., are dominated) and the ICER is not calculated. The ceiling ratio (λ) is used as a cut-off point to decide whether an intervention is deemed cost-effective or cost-ineffective. Graphically, it represents a line through the origin of the CEP with a slope equal to λ and divides the CEP into an acceptable and rejectable area (Figure 2a) [7,26]. Note that the interpretations of λ in the NE quadrant and SW quadrant are different. In the NE quadrant λ represents the decision maker’s maximum WTP per health outcome whereas in the SW quadrant it represents the decision maker’s minimum WTA compensation for relinquishing health outcomes. Furthermore, note that the ICER has a different valuation in the NE quadrant and SW quadrant [27]. Assuming a ceiling ratio λ of 1000 $ per QATY (Quality-Adjusted Tooth-Year), we would accept program A (Figure 2a) with an ICER of 500 $/QATY since it is less than the maximum WTP per QATY. On the other hand, we would reject program B (Figure 2a) with the same ICER of 500 $/QATY since it is less than the minimum monetary compensation of 1000 $/QATY required to accept health loss (WTA). In other words, the decision rule in the NE quadrant of the CEP is mirrored in the SW quadrant [15].

The classical decision rule of assuming the same ceiling ratio λ in the NE and SW quadrant is not supported by empirical evidence [7,16,17,18,19]. A disparity between WTA and WTP results in a kinked threshold line as shown in Figure 2b where a WTA/WTP ratio of 2 is assumed [7]. This leads to a smaller acceptable area on the CEP, depicted by the grey shaded area in Figure 2b. The higher the WTA/WTP disparity, the smaller the acceptable area becomes in the SW quadrant of the CEP. This has important implications for stochastic cost-effectiveness analysis where the joint distribution of incremental costs and effects ƒ(ΔC,ΔE) may extend into the SW quadrant of the CEP.

**Figure 3:**
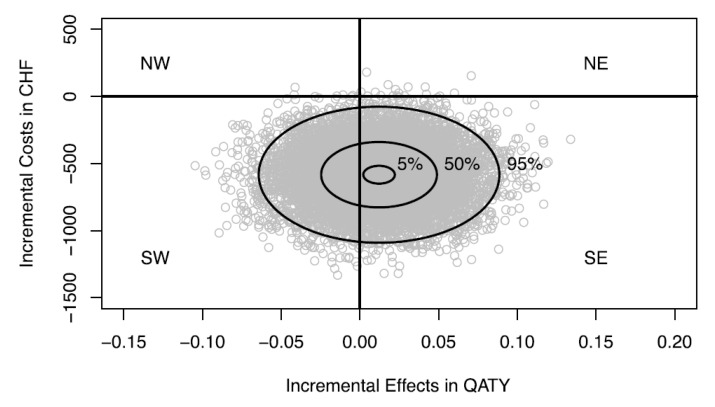
Joint distribution of incremental costs and effects on the cost-effectiveness plane. Mean ΔC −584 Swiss Francs, mean ΔE 0.01 QATY (Quality-Adjusted Tooth-Year) from Zitzmann et al. (2013) [28]. The 95%, 50% and 5% credible ellipses are drawn. NW, North-West; SW, South-West; NE, North-East; SE, South-East.

**Figure 4:**
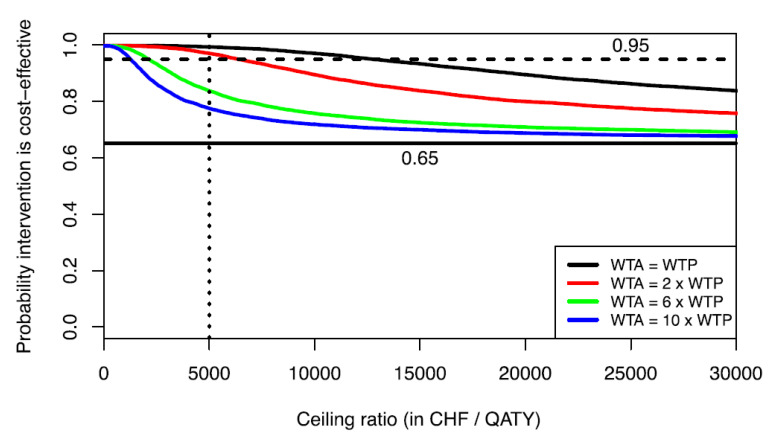
Cost-effectiveness acceptability curves for different WTA/WTP ratios. Horizontal line at 0.65 indicates probability of dominance; horizontal line at 0.95 indicates threshold line for hypothesis testing within a frequentist framework.

As a practical example to demonstrate our conceptional point, data from a published stochastic cost-effectiveness analysis comparing single tooth implant restorations vs. 3-unit fixed dental protheses are used [28]. Over a time horizon of three years, anterior implants were dominant with cost savings of 584 CHF (Swiss Francs) and a gain in 0.01 QATY on average. The joint distribution ƒ(ΔC,ΔE) of the second-order Monte Carlo simulation using 10,000 samples from input distributions and 10,000 runs per sample to account for sample variation is shown in Figure 3. As can be seen, ƒ(ΔC,ΔE) substantially covers the SW quadrant of the CEP. The probability of dominance (i.e., SE quadrant) is 0.65 (Figure 3 and horizontal line in Figure 4). We have recalculated the cost-effectiveness acceptability curves, which estimate the probability that anterior implants are cost-effective compared to fixed dental prostheses as a function of the ceiling ratio λ. Figure 4 shows the cost-effectiveness acceptability curves for different WTA/WTP ratios as suggested by Severens et al. [16]. The probability that anterior implants are cost-effective compared to fixed dental prostheses decreases as the WTA/WTP ratio increases. This reflects a smaller proportion of the joint distribution ƒ(ΔC,ΔE) in the acceptable area of the SW quadrant of the CEP as the ceiling ratio increases. 

Although it has been argued that the interpretation of cost-effectiveness acceptability curves is Bayesian in nature [29], i.e., the probability that the intervention is cost-effective given λ, Löthgren and Zethraeus (2000) have shown that the cost-effectiveness acceptability curve also has an interpretation within a frequentist framework in that it estimates the probability that the incremental net monetary benefit (INMB), defined as

INMB = ΔE λ − ΔC(2)

is positive [30]. The INMB is a linear transformation of the cost-effectiveness ratio and has been introduced to facilitate statistical analysis [9,14]. For a given ceiling ratio λ, a treatment alternative would be preferred if the INMB would be positive, i.e., would offer more net benefits than the comparator. At a significance level of 5%, one can therefore test the null hypothesis that the INMB is non-positive using the cost-effectiveness acceptability curve. In Figure 4, one can see that at a ceiling ratio of $5000 per QATY (vertical dotted line), the null-hypothesis that INMB is non-positive is rejected (*p* < 0.05), assuming no disparity between WTA and WTP, since the cost-effectiveness acceptability curve lies above the threshold probability level of 0.95 (horizontal dashed line). However, when a WTA/WTP ratio of 6 is assumed, then the cost-effectiveness acceptability curve falls below the 0.95 threshold probability level, and one would accept the null hypothesis that the INMB is non-positive (*p* > 0.05). This example clearly shows the impact of a WTA/WTP disparity on cost-effectiveness analysis in dental health care. 

## 5. Discussion

In the present paper, we have shown (i) that there is limited evidence and a knowledge gap about WTA/WTP ratios for dental interventions, (ii) that the WTA/WTP ratio may be higher for implant rehabilitations than for public health interventions, and (iii) that the WTA/WTP ratio may impact the results of a stochastic cost-effectiveness analysis and should therefore be part of the economic evaluation.

The WTA/WTP ratios found in the present review range from 2.58 for discontinuing water fluoridation to 5.12 for mandibular implant overdentures. These estimates are in line with those reported for other medical and safety interventions. In their review, Horowitz and McConnell (2002) found a mean WTA/WTP ratio of 10.06 for health and safety studies [18]. In a more recent updated meta-analysis, Tunçel and Hammitt (2014) report a mean WTA/WTP ratio of 5.09 for health and safety goods [17]. O’Brien et al. (2002) reported separate WTA/WTP ratios for health studies ranging from 1.9 to 6.4, and for safety studies ranging from 1.1 to 3.6 [7]. Rotteveel et al. (2020) conducted a meta-analysis of WTA/WTP ratios for health care goods and services using individual participant data and found a mean ratio of 1.70 [19]. The size of the WTA/WTP ratio may depend on the health care good and service provided and the size of the change valued and may be larger for larger changes in health [31]. This may also explain the much smaller WTA/WTP ratio for water fluoridation than for implant rehabilitations.

While the disparity between WTA and WTP is a consistent observation in empirical studies, several reasons have been suggested that may explain that losses are valued higher than equivalent gains. These include income effects and transaction costs, the absence of substitutes and endowment effects (i.e., loss aversion) [7,17,18]. Since WTP is constrained by income, whereas compensation demanded to forgo a good (WTA) is not, WTA-WTP-disparity may increase for more expensive services such as oral implant treatment. Transaction costs relates to the costs associated with buying or selling a good [32]. To the extent that transaction costs affects buyers and sellers differently, a WTA-WTP-disparity will result. Whereas no tangible transaction costs may be obvious for water fluoridation or implant surgery, intangible transaction costs may result as a hypothetical disutility of removing implants in patients wearing mandibular implant overdentures, leading to a higher WTA/WTP ratio. The difference between WTA and WTP is usually small when a perfect substitute is available for the respective service or good [33]. When perfect substitutes are not available that money can buy, the WTA/WTP ratio will be larger. This is typically the case for non-market goods such as oral health care and even more so for complex surgical and prosthodontic interventions. Endowment effect relates to loss aversion in that a desirable good is more valuable if it is part of a person’s endowment than when it is not [34]. This will result in an asymmetric valuation of gains and losses and hence in a higher WTA/WTP ratio. Whereas the reasons to explain a WTA-WTP-disparity are subject of many investigations, the presence of a WTA-WTP-disparity for virtually all health care goods and services is indisputable [17,18,19,35].

Our study has several limitations. First, we were only able to include two studies and only one for individual participant analysis. However, this is not a limitation of the study per se but indicates a knowledge gap and a lack of evidence in the dental literature. It therefore suggests a potential area for future research to improve the allocation of resources that is more in line with the preferences of patients and decision makers over uncertain costs and effect. Second, the WTA/WTP ratios estimated in our systematic review may be subject to considerable uncertainty due to the small sample size of the included studies, emphasizing the importance of further studies with larger sample sizes. However, this limitation can be addressed by conducting a sensitivity analysis of different WTA/WTP ratios in an economic evaluation of dental interventions as suggested by Severens et al. (2005) [16]. Third, the administration format of WTA and WTP questions in contingent valuation may influence the number of missing values or protest answers. In the one included study for individual participant analysis, WTA questions were open-ended leading to a higher number of missing values [24]. Payment cards or dichotomous-choice questions may lead to a higher percentage of answers revealing true preferences [36]. However, we used multiple imputation with chained equations to impute missing WTA values which performs well also with small datasets [23].

## 6. Conclusions

To the best of our knowledge, this is the first study on WTA/WTP disparity in dentistry. There is a lack of evidence of WTA/WTP ratios in oral health care, with only two included studies, reporting a higher WTA/WTP ratio for implant rehabilitations than for dental public health interventions. A WTA-WTP disparity inflates the cost-effectiveness of dental interventions when there is a considerable risk of both lower costs and health outcomes. We recommend that in these cases the results of cost-effectiveness analyses in dentistry are reported using different WTA/WTP ratios in a sensitivity analysis.

## Figures and Tables

**Table 1:** Studies included for estimating the WTA /WTP ratio.

Publication	Sendi et al., 2017 [24]	Dixon and Shackley, 1999 [25]
Intervention	Mandibular implant overdentures	Discontinuing water fluoridation
Country	Switzerland	United Kingdom
Study design	Contingent valuation	Contingent valuation
Elicitation method	Iterative bidding (WTP) Open-ended (WTA)	Payment cards
Comparison group	Within subject design	Within subject design
Mean WTP	4606 CHF (95% CI: 3547–6560) ^1^ (*n* = 15)	29.38 GBP (*n* = 8)
Mean WTA	33,500 CHF (95% CI: 9000–72,000) ^1^ (*n* = 5)	76.00 GBP (*n* = 5)
Mean WTA/WTP ratio	5.31 (95% CI: 2.34–8.20) ^1^ (*n* = 5)	N.A.
WTA/WTP ratio (ratio of means)	7.27	2.58
Mean WTA/WTP ratio (mean ratio using MICE) ^2^	5.12 (95% CI: 2.61–7.62)	N.A.

WTA denotes Willingness to Accept; WTP denotes Willingness to Pay; CHF denotes Swiss Francs; GBP denotes British Pounds; ^1^ 95% confidence intervals were estimated using bias corrected and accelerated bootstrapping (10,000 samples); ^2^ MICE indicates multiple imputation with chained equations; pooled estimates are based on 1000 imputed datasets.

## Supplementary Materials

The following are available online at https://www.mdpi.com/2227-9032/8/3/301/s1, Supplementary Material: Eligible papers excluded.
