# Supplementary material for: A Systematic Review of WTA-WTP Disparity for Dental Interventions and Implications for Cost-Effectiveness Analysis
Source: Healthcare (Basel). 2020 Aug 26;8(3):301. doi: 10.3390/healthcare8030301 (PMC7550993; doi:10.3390/healthcare8030301)
Supplement: Supplementary file 1 [file healthcare-08-00301-s001.pdf]

Eligible papers excluded (n = 52, reason: WTA not assessed)

Al Garni, B.; Pani, S. C.; Almaaz, A.; Al Qeshtaini, E.; Abu-Haimed, H.; Al Sharif, K., Factors affecting the willingness to pay for implants: A study of patients in Riyadh, Saudi Arabia. *Dent Res J (Isfahan)* **2012,** *9*, 719-24.

Atanasov, N.; Stoyanova, R.; Alexandrova, M., Possibilities to Model Patients' Preferences and Their Willingness to Pay for a Molar Treatment. *Dent Med Probl* **2016,** *53*, 41-49.

Atchison, K. A.; Gironda, M. W.; Black, E. E.; Schweitzer, S.; Der-Martirosian, C.; Felsenfeld, A.; Leathers, R.; Belin, T. R., Baseline characteristics and treatment preferences of oral surgery patients. *J Oral Maxillofac Surg* **2007,** *65*, 2430-7.

Augusti, D.; Augusti, G.; Re, D., Prosthetic restoration in the single-tooth gap: patient preferences and analysis of the WTP index. *Clin Oral Implants Res* **2014,** *25*, 1257-1264.

Balevi, B.; Shepperd, S., The management of an endodontically abscessed tooth: patient health state utility, decision-tree and economic analysis. *BMC Oral Health* **2007,** *7*, 17.

Bech, M.; Kjaer, T.; Lauridsen, J., Does the Number of Choice Sets Matter? Results from a Web Survey Applying a Discrete Choice Experiment. *Health Economics* **2011,** *20*, 273-286.

Berendsen, J.; Bonifacio, C.; van Gemert-Schriks, M.; van Loveren, C.; Verrips, E.; Duijster, D., Parents' willingness to invest in their children's oral health. *J Public Health Dent* **2018,** *78*, 69-77.

Birch, S.; Sohn, W.; Ismail, A. I.; Lepkowski, J. M.; Belli, R. F., Willingness to pay for dentin regeneration in a sample of dentate adults. *Community Dent Oral Epidemiol* **2004,** *32*, 210-6.

Christell, H.; Gullberg, J.; Nilsson, K.; Heidari Olofsson, S.; Lindh, C.; Davidson, T., Willingness to pay for osteoporosis risk assessment in primary dental care. *Health Econ Rev* **2019,** *9*, 14.

Emami, E.; Alesawy, A.; de Grandmont, P.; Cerutti-Kopplin, D.; Kodama, N.; Menassa, M.; Rompre, P.; Durand, R., A within-subject clinical trial on the conversion of mandibular two-implant to three-implant overdenture: Patient-centered outcomes and willingness to pay. *Clin Oral Implants Res* **2019,** *30*, 218-228.

Ethier, M. C.; Regier, D. A.; Tomlinson, D.; Judd, P.; Doyle, J.; Gassas, A.; Naqvi, A.; Sung, L., Perspectives toward oral mucositis prevention from parents and health care professionals in pediatric cancer. *Support Care Cancer* **2012,** *20*, 1771-7.

Farronato, G.; Re, D.; Augusti, G.; Butti, A.; Augusti, D., Biomimetic orthodontic treatments: preferences of adult patients and analysis of the Willingness-To-Pay index. *Dent Cadmos* **2016,** *84*, 408-417.

Feu, D.; Catharino, F.; Duplat, C. B.; Capelli Junior, J., Esthetic perception and economic value of orthodontic appliances by lay Brazilian adults. *Dental Press J Orthod* **2012,** 17, 102-14.

Goldstein, M.; Sumner, W.; Littenberg, B., Preferences and willingness to pay for painless dental laser drill. *Med Decis Making* **1998,** *18*, 457-457.

Halvorsen, B.; Willumsen, T., Willingness to pay for dental fear treatment. Is supplying dental fear treatment socially beneficial? *Eur J Health Econ* **2004,** *5*, 299-308.

Harris, R.; Lowers, V.; Laverty, L.; Vernazza, C.; Burnside, G.; Brown, S.; Ternent, L., Comparing how patients value and respond to information on risk given in three different forms during dental check-ups: the PREFER randomised controlled trial. *Trials* **2020,** *21*, 21.

Koberlein, J.; Klingenberger, D., [Foreign dentures and dental tourism--willingness-to-pay and factors influencing the demand for foreign dental prosthesis in Germany]. *Gesundheitswesen* **2011,** *73*, e111-8.

Leung, K. C.; McGrath, C. P., Willingness to pay for implant therapy: a study of patient preference. *Clin Oral Implants Res* **2010,** *21*, 789-93.

Matthews, D.; Rocchi, A.; Gafni, A., Putting your money where your mouth is: willingness to pay for dental gel. *Pharmacoeconomics* **2002,** *20*, 245-55.
Matthews, D.; Rocchi, A.; Wang, E. C.; Gafni, A., Use of an interactive tool to assess patients' willingness-to-pay. *J Biomed Inform* **2001,** *34*, 311-20.
Matthews, D. C.; Birch, S.; Gafni, A.; DiCenso, A., Willingness to pay for periodontal therapy: development and testing of an instrument. *J Public Health Dent* **1999,** *59*, 44-51.
McKenna, G.; Tada, S.; Woods, N.; Hayes, M.; DaMata, C.; Allen, P. F., Tooth replacement for partially dentate elders: A willingness-to-pay analysis. *J Dent* **2016,** *53*, 51-6.
Nair, R.; Yee, R., Differences in willingness to pay for an extraction, a filling, and cleaning teeth at various levels of oral health-related quality of life, as measured by oral impacts on daily performance, among older adults in Singapore. *Singapore Dent J* **2016,** *37*, 2-8.
Ndambiri, H.; Rotich, E., Valuing excess fluoride removal for safe drinking water in Kenya. *Water Policy* **2018,** *20*, 953-965.
Nyamuryekung'e, K. K.; Lahti, S. M.; Tuominen, R. J., Patients' willingness to pay for dental services in a population with limited restorative services. *Community Dent Health* **2018,** *35*, 167-172.
Oscarson, N.; Lindholm, L.; Kallestal, C., The value of caries preventive care among 19-year olds using the contingent valuation method within a cost-benefit approach. *Community Dent Oral Epidemiol* **2007,** *35*, 109-17.
Pavlova, M.; Groot, W.; Van Merode, G., Willingness and ability of Bulgarian consumers to pay for improved public health care services. *Appl Econ* **2004,** *36*, 1117-1130.
Ramsay, C. R.; Clarkson, J. E.; Duncan, A.; Lamont, T. J.; Heasman, P. A.; Boyers, D.; Goulao, B.; Bonetti, D.; Bruce, R.; Gouick, J.; Heasman, L.; Lovelock-Hempleman, L. A.; Macpherson, L. E.; McCracken, G. I.; McDonald, A. M.; McLaren-Neil, F.; Mitchell, F. E.; Norrie, J. D.; van der Pol, M.; Sim, K.; Steele, J. G.; Sharp, A.; Watt, G.; Worthington, H. V.; Young, L., Improving the Quality of Dentistry (IQuaD): a cluster factorial randomised controlled trial comparing the effectiveness and cost-benefit of oral hygiene advice and/or periodontal instrumentation with routine care for the prevention and management of periodontal disease in dentate adults attending dental primary care. *Health Technol Assess* **2018,** *22*, 1-144.
Re, D.; Augusti, G.; Battaglia, D.; Gianni, A. B.; Augusti, D., Is a new sonic toothbrush more effective in plaque removal than a manual toothbrush? *Eur J Paediatr Dent* **2015,** *16*, 13-8.
Re, D.; Ceci, C.; Cerutti, F.; Fabbro, M. D.; Corbella, S.; Taschieri, S., Natural tooth preservation versus extraction and implant placement: patient preferences and analysis of the willingness to pay. *Br Dent J* **2017,** *222*, 467-471.
Re, D.; Del Fabbro, M.; Karanxha, L.; Augusti, G.; Augusti, D.; Fessi, S.; Taschieri, S., Minimally-invasive dental anesthesia: Patients' preferences and analysis of the willingness-to-pay index. *J Investig Clin Dent* **2018,** *9*.
Rosvall, M. D.; Fields, H. W.; Ziuchkovski, J.; Rosenstiel, S. F.; Johnston, W. M., Attractiveness, acceptability, and value of orthodontic appliances. *Am J Orthod Dentofacial Orthop* **2009,** *135*, 276 e1-12; discussion 276-7.
Rutkowski, J. L., What Is the Monetary Value of Dental Implant Treatments? *J Oral Implantol* **2019,** *45*, 171.
Sever, I.; Verbic, M.; Klaric Sever, E., Estimating Attribute-Specific Willingness-to-Pay Values from a Health Care Contingent Valuation Study: A Best-Worst Choice Approach. *Appl Health Econ Health Policy* **2020,** *18*, 97-107.
Sever, I.; Verbic, M.; Sever, E. K., Valuing the delivery of dental care: Heterogeneity in patients' preferences and willingness-to-pay for dental care attributes. *J Dent* **2018,** *69*, 93-101.
Sever, I.; Verbic, M.; Sever, E. K., Estimating willingness-to-pay for health care: A discrete choice experiment accounting for non-attendance to the cost attribute. *J Eval Clin Pract* **2019,** *25*, 843-849.

Smith, A. S.; Cunningham, S. J., Which factors influence willingness-to-pay for orthognathic treatment? *Eur J Orthod* **2004,** *26*, 499-506.
Srivastava, A.; Esfandiari, S.; Madathil, S. A.; Birch, S.; Feine, J. S., Willingness to Pay for Mandibular Overdentures: A Societal Perspective. *JDR Clin Trans Res* **2020,** *5*, 30-39.
Srivastava, A.; Feine, J. S.; Esfandiari, S., Are people who still have their natural teeth willing to pay for mandibular two-implant overdentures? *J Investig Clin Dent* **2014,** *5*, 117-24.
Stone, S. J.; McCracken, G. I.; Heasman, P. A.; Staines, K. S.; Pennington, M., Cost-effectiveness of personalized plaque control for managing the gingival manifestations of oral lichen planus: a randomized controlled study. *J Clin Periodontol* **2013,** *40*, 859-67.
Tamaki, Y.; Nomura, Y.; Teraoka, K.; Nishikahara, F.; Motegi, M.; Tsurumoto, A.; Hanada, N., Characteristics and willingness of patients to pay for regular dental check-ups in Japan. *J Oral Sci* **2004,** *46*, 127-33.
Tianviwat, S.; Chongsuvivatwong, V.; Birch, S., Prevention versus cure: measuring parental preferences for sealants and fillings as treatments for childhood caries in Southern Thailand. *Health Policy* **2008,** *86*, 64-71.
Tianviwat, S.; Chongsuvivatwong, V.; Birch, S., Different dental care setting: does income matter? *Health Econ* **2008,** *17*, 109-18.
Tianviwat, S.; Chongsuvivatwong, V.; Birch, S., Optimizing the mix of basic dental services for Southern Thai schoolchildren based on resource consumption, service needs and parental preference. *Community Dent Oral Epidemiol* **2009,** *37*, 372-80.
Tuominen, R., Evaluation of three methods assessing the relative value of a dental program. *Acta Odontol Scand* **2008,** *66*, 82-7.
van Steenberghe, D.; Bercy, P.; De Boever, J.; Adriaens, P.; Geers, L.; Hendrickx, E.; Adriaenssen, C.; Rompen, E.; Malmenas, M.; Ramsberg, J., Patient evaluation of a novel non-injectable anesthetic gel: a multicenter crossover study comparing the gel to infiltration anesthesia during scaling and root planing. *J Periodontol* **2004,** *75*, 1471-8.
Vermaire, J. H.; van Exel, N. J.; van Loveren, C.; Brouwer, W. B., Putting your money where your mouth is: parents' valuation of good oral health of their children. *Soc Sci Med* **2012,** *75*, 2200-6.
Vernazza, C. R.; Steele, J. G.; Whitworth, J. M.; Wildman, J. R.; Donaldson, C., Factors affecting direction and strength of patient preferences for treatment of molar teeth with nonvital pulps. *Int Endod J* **2015,** *48*, 1137-46.
Vernazza, C. R.; Wildman, J. R.; Steele, J. G.; Whitworth, J. M.; Walls, A. W.; Perry, R.; Matthews, R.; Hahn, P.; Donaldson, C., Factors affecting patient valuations of caries prevention: Using and validating the willingness to pay method. *J Dent* **2015,** *43*, 981-8.
Walshaw, E. G.; Adam, N. I.; Palmeiro, M. L.; Neves, M.; Vernazza, C. R., Patients' and Parents' Valuation of Fluoride. *Oral Health Prev Dent* **2019,** *17*, 211-218.
Widstrom, E.; Seppala, T., Willingness and ability to pay for unexpected dental expenses by Finnish adults. *BMC Oral Health* **2012,** *12*, 35.
Willumsen, T., Patient' willingness to pay for treatment of dental phobia. *J Dent Res* **2000,** *79*, 145-145.

Eligible paper excluded (n = 1, reason: WTP and WTA assessed, but WTA not published)

Esfandiari, S.; Lund, J. P.; Penrod, J. R.; Savard, A.; Thomason, J. M.; Feine, J. S., Implant overdentures for edentulous elders: study of patient preference. *Gerodontology* **2009,** *26*, 3-10.
